# Complete radiation-free, transthoracic echocardiography-guided, leadless pacemaker implantation

**DOI:** 10.1093/ehjcr/ytaf069

**Published:** 2025-02-19

**Authors:** Wei Hua, Hao Huang, Xuhua Chen, Xiangbin Pan

**Affiliations:** State Key Laboratory of Cardiovascular Disease, National Center for Cardiovascular Diseases, Chinese Academy of Medical Sciences, Peking Union Medical College, Fuwai Hospital, No. 167 North Lishi Road, Xicheng District, Beijing 100037, China; State Key Laboratory of Cardiovascular Disease, National Center for Cardiovascular Diseases, Chinese Academy of Medical Sciences, Peking Union Medical College, Fuwai Hospital, No. 167 North Lishi Road, Xicheng District, Beijing 100037, China; State Key Laboratory of Cardiovascular Disease, National Center for Cardiovascular Diseases, Chinese Academy of Medical Sciences, Peking Union Medical College, Fuwai Hospital, No. 167 North Lishi Road, Xicheng District, Beijing 100037, China; State Key Laboratory of Cardiovascular Disease, National Center for Cardiovascular Diseases, Chinese Academy of Medical Sciences, Peking Union Medical College, Fuwai Hospital, No. 167 North Lishi Road, Xicheng District, Beijing 100037, China

## Case description

**Figure 1:**
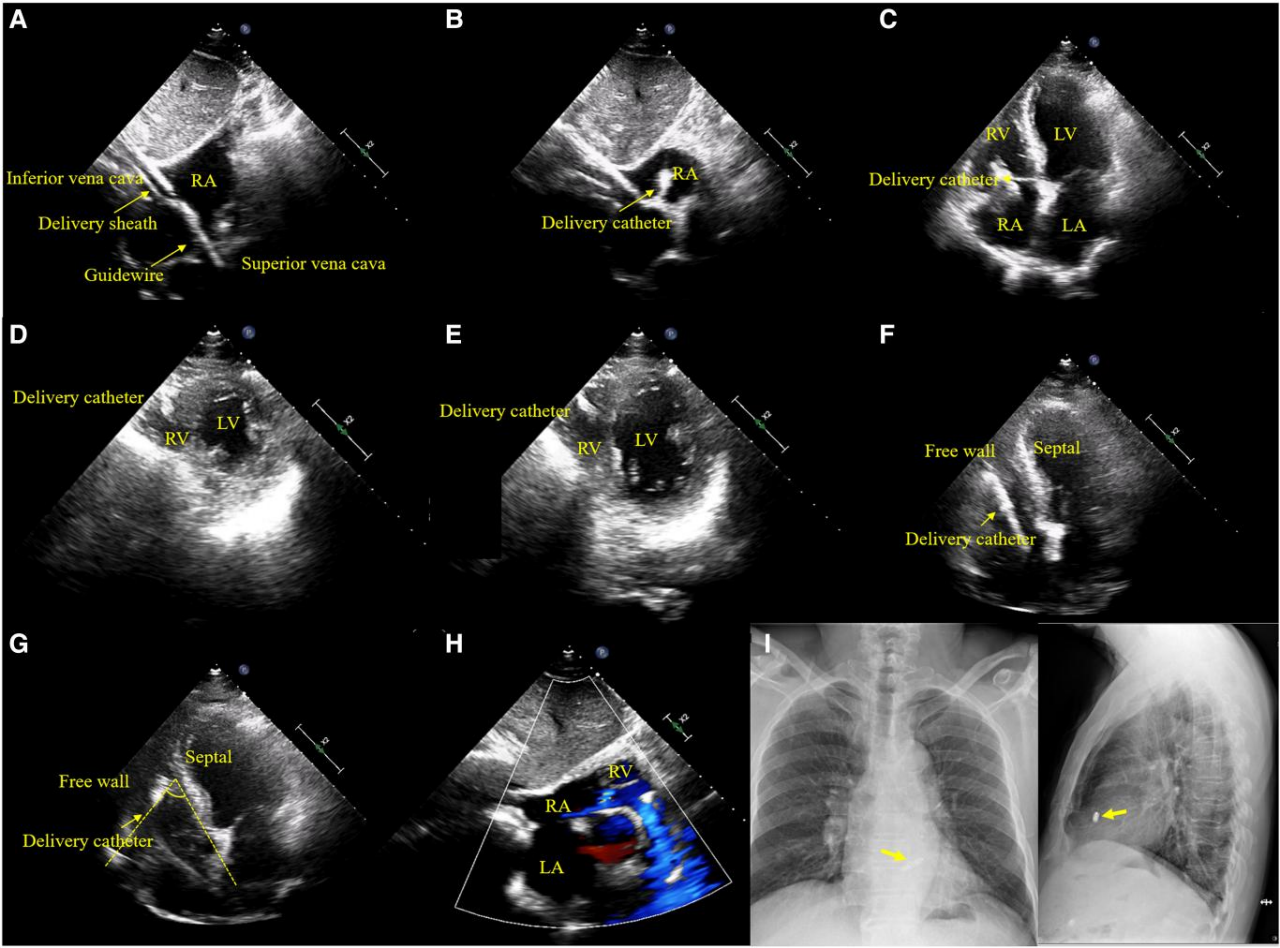
(*A*) Guidewire was introduced to the entry of the superior vena cava, followed by delivery sheath entering the inferior vena cava. (*B*) Transcatheter pacing system was advanced to the right atrium. (*C*) Delivery catheter crossed the tricuspid valve. Delivery catheter in the right ventricle from the parasternal short-axis view at the apical (*D*) and papillary muscle (*E*) levels. (*F*, *G*) Delivery system was moved from the right ventricular-free wall towards the interventricular septum. Notice that a pronounced curve was pushed against the right ventricular septum before fixation in *G*. (*H*) The tricuspid valve tricuspid regurgitation from the subxiphoid view. (*I*) Chest X-ray on the second day from anteroposterior and left lateral view. LA, left atrium; LV, left ventricle, RA, right atrium; RV, right ventricle.

A 74-year-old male was referred for pacemaker implantation due to intermittent high-grade conduction block, with a maximum R–R interval of 2.95 s noted after atrial fibrillation ablation. The patient was opted for Micra™ leadless pacemaker implantation completely guided by transthoracic echocardiography (TTE), using a Philips EPIQ ultrasound system (Philips, Amsterdam, the Netherlands) and X5-1 transducer. Hydrophilic guidewire was initially introduced to the entry of the superior vena cava (*Figure 1A*). The Micra AV transcatheter system was advanced to the right atrium (*Figure 1B*; Supplementary material online, *Video S1*). Transthoracic echocardiography clearly visualized the pacing system crossing the tricuspid valve from the apical four-chamber view (A4C; *Figure 1C*; Supplementary material online, *Video S2*), and advancing to the right ventricular (RV) inflow tract from the parasternal short-axis view (PSAX; *Figure 1D* and *E*; Supplementary material online, *Video S3*). The RV outflow tract was ruled out, before the Micra moved from the RV-free wall towards the interventricular septum, with the delivery system pushed into a pronounced curve by the RV septum. (*Figure 1F* and *G*; Supplementary material online, *Video S4*). The device was then deployed. The instant capture threshold was 1.0 V/0.24 ms; the sensed R wave was 5.8 mV; and the impedance was 957 ohms. Tricuspid regurgitation was unaffected (*Figure 1H*; Supplementary material online, *Video S5*). The chest X-ray showed the Micra was implanted in the high septal region and was securely fixed (*Figure 1I*). The device parameters remained stable in 3-month follow-ups.

This case demonstrates that 2D TTE can guide the entire implantation of a leadless pacemaker without fluoroscopy. The echo windows in the chest and the abdomen should be appropriately assessed. The A4C provides better demonstration of the sheath's trajectory and direction against the septum, while the PSAX confirms the depth of the sheath within the RV inflow tract. A gooseneck sign before the release and favourable acute parameters ensured secure fixation. Further studies are needed to evaluate the long-term outcomes and broader applicability.

## Supplementary Material

ytaf069_Supplementary_Data

## Data Availability

Data sharing is not applicable to this report because no data sets were generated or analysed for this case.

## Notes

**Consent:** The authors confirm written consent for the submission and publication of this case report including images. The written consent form was compliant with the COPE guidelines.

**Funding:** None declared.

